# The Cardioprotective Effect of Hypertonic Saline Is Associated with Inhibitory Effect on Macrophage Migration Inhibitory Factor in Sepsis

**DOI:** 10.1155/2013/201614

**Published:** 2013-11-26

**Authors:** Yi-Li Wang, Kwok-Keung Lam, Pao-Yun Cheng, Ching-Wen Kung, Shu-Ying Chen, Chun-Chih Chao, Hwong-Ru Hwang, Ming-Ting Chung, Yen-Mei Lee

**Affiliations:** ^1^Graduate Institute of Life Sciences, National Defense Medical Center, Taipei, Taiwan; ^2^Department of Pharmacology, Taipei Medical University, Taipei, Taiwan; ^3^Department of Anesthesiology, Catholic Mercy Hospital, Hsinchu, Taiwan; ^4^Department of Physiology & Biophysics, National Defense Medical Center, Taipei, Taiwan; ^5^Department of Nursing, Tzu Chi College of Technology, Hualien, Taiwan; ^6^Department of Nursing, HungKuang University, Taichung, Taiwan; ^7^Department of Pharmacology, National Defense Medical Center, 160 Min-Chuan E. Road, Taipei 114, Taiwan; ^8^Division of Cardiology, Department of Medicine, Kaohsiung Veterans General Hospital, Kaohsiung, Taiwan; ^9^Center for Reproductive Medicine, Department of Obstetrics and Gynecology, Chi-Mei Medical Center, 901 Chung Hwa Road, Tainan 710, Taiwan; ^10^Chia Nan University of Pharmacy & Science, Tainan, Taiwan

## Abstract

Sepsis can cause myocardial dysfunction, which contributes to the high mortality of sepsis. Hypertonic saline (HS) has been reported to increase myocardial contractility in sepsis. In the present study, mechanisms of action of HS resuscitation (4 mL of 7.5% NaCl per kilogram) on cardiac function have been evaluated in septic rats. HS was administered 1 h after LPS (10 mg/kg, i.v.) challenge. The mean arterial blood pressure significantly decreased 4 h after LPS challenge, and septic shock was observed at the end of experiment (6 h). Posttreatment with HS prevented hypotension caused by LPS and significantly improved cardiac function, evidenced by increases in left ventricular developed pressure, mean +dP/dt and −dP/dt. The amplitude of electrical-stimulated intracellular Ca^2+^ transient in isolated single cardiomyocytes was significantly reduced after 6 h LPS insult, which was recovered by HS. In addition, LPS resulted in significant increases in neutrophil myeloperoxidase activity, macrophage migration inhibitory factor (MIF), and NF-*κ*B phospho-p65 protein levels in myocardium at 6 h, which were significantly attenuated by HS. In conclusion, HS improved myocardial contractility and prevented circulatory failure induced by endotoxemia, which may attribute to improvement of intracellular calcium handling process and inhibitory effects on neutrophil infiltration and MIF production in hearts.

## 1. Introduction

Multiple organ failure is a leading cause of mortality in sepsis, and myocardial depression is the most common organ dysfunction. Sepsis-induced cardiac dysfunction is characterized by decreased myocardial contractility, impaired ventricular response to fluid therapy, and ventricular dilatation [1]. Fluid resuscitation is one of the first-line cornerstone therapies and to support the cardiac function in severe sepsis [2]. Isotonic fluid (Ringer's lactate or normal saline [0.9% NaCl]) administration can restore the body fluid and microvascular perfusion. In clinical therapy, small volume of hypertonic saline (HS) [7.5% NaCl] recovers hemodynamic variables and effective circulating volume in hemorrhagic shock [3]. The beneficial effect of HS is associated with its anti-inflammatory effect, evidenced by inhibition of neutrophil activation and infiltration in lungs [4]. Neutrophil activation can release cytokines, reactive oxygen species, and enzymes, resulting in injuries of organs and tissues [5]. Furthermore, HS can ameliorate organ dysfunction in severe sepsis caused by cecal ligation and puncture (CLP), which is mediated via its antioxidant and anti-inflammatory effects [6]. Recently, HS has been revealed to prevent early myocardial dysfunction and to reduce myocardial apoptosis [7].

Macrophage migration inhibitory factor (MIF) is one of the important factors in sepsis. MIF is ubiquitously expressed in both immune and nonimmune cells including various peripheral tissues. MIF can recruit immune cells (macrophages, eosinophils, basophils, neutrophils) to the site of inflammation, leading to amplify the production of various proinflammatory cytokines and mediators such as IL-1*β*, TNF-*α*, IFN-*γ*, IL-17, and nitric oxide (NO) [8]^.^ After injection of LPS in rodents, MIF protein was released from several organs, such as lung, liver, kidney, adrenal and pituitary gland, spleen, and skin [9]. MIF protein expression also significantly increased in hearts of septic mice [10, 11]. Recently, it has been demonstrated that HS reduces the levels of MIF in LPS-induced macrophage cell line [12]. MIF neutralization by anti-MIF antibody can reverse endotoxin-induced myocardial dysfunction in rats [13]. Therefore, we examined whether neutrophil infiltration and MIF expression are involved in the cardioprotective effect of HS in a conscious rat model of LPS-induced sepsis.

Ca^2+^ influx through the L-type Ca^2+^ channel (LTCCs) of sarcolemma of myocardium during an action potential initiates contraction of the cardiac myocytes. Ca^2+^ current subsequently triggers a larger Ca^2+^ release from the sarcoplasmic reticulum (SR) via ryanodine receptors, resulting in elevation of intracellular Ca^2+^ concentration ([Ca^2+^]_i_), and providing Ca^2+^ for the excitation-contraction coupling [14]. In this study, we further measured the amplitude of [Ca^2+^]_i_ transients in isolated single cardiomyocytes to evaluate whether HS can preserve [Ca^2+^]_i_ handling capacity to improve cardiac contractile function during sepsis.

## 2. Materials and Methods

### 2.1. Experimental Animals

Male Wistar rats (10–12 weeks old, 280–300 g) were used and purchased from the National Laboratory Animal Breeding and Research Center of the National Science Council, Taiwan. Handling of the animals conforms to the *Guide for the Care and Use of Laboratory Animals*, published by the National Institutes of Health, USA (NIH publication number 85-23, revised in 1996). All animal cares and experimental protocols in this study were approved by the Animal Care and Use Committee of National Defense Medical Center, Taipei, Taiwan. Animals were housed under a 12 h light-dark cycle room with an ambient temperature of 22 ± 1°C and humidity of 50 ± 5%. The animal preparation for anesthetization and cannulation of blood vessels were performed as described previously [15].

### 2.2. Experimental Groups

The experiments of sepsis were performed on conscious rats, which has been reported to be a clinically relevant sepsis model [16] and avoids the interference of anesthetics with cytokine release [17]. Animals were divided into four groups: (1) sham (normal saline, 0.9% NaCl, 4 mL/kg, intravenously), *n* = 6; (2) sham + HS (7.5% NaCl, 4 mL/kg, intravenously), *n* = 6; (3) LPS: rats were treated with* Escherichia coli* LPS 10 mg/kg (intravenous infusion for 10 min). One hour after LPS administration, 0.9% NaCl (4 mL/kg, 300 mosmole/L) was given intravenously, *n* = 10; (4) LPS + HS: rats were treated with* Escherichia coli* LPS 10 mg/kg (intravenous infusion for 10 min). One hour after LPS administration, 7.5% NaCl (4 mL/kg, 2400 mosmole/L) was given, *n* = 10. Normal saline and HS were infused with a rate of 0.2 mL/min [18, 19]. At 0, 1, 2, 4, and 6 h after LPS infusion, the changes in hemodynamics (blood pressure and heart rate), hepatic function index (i.e., alanine aminotransferase (ALT), aspartate aminotransferase (AST)), cell toxicity index (i.e., lactate dehydrogenase (LDH)), and renal function index (creatinine (CRE)), as well as the plasma levels of sodium, potassium, and calcium ion concentration were examined. Six hours after LPS infusion, animals were sacrificed and hearts were collected immediately.

### 2.3. Isolated Heart Preparation and Left Ventricular Pressure Recording

The preparation for heart isolation and measurement of cardiac contractility were performed as described previously [15]. Hearts were isolated 6 h after LPS administration and mounted on the Langendorff apparatus (ML785B2 Langendorff System Bundle, AD instruments). The left ventricular developed pressure (LVDP) and the mean rates of contraction (+dP/dt) and relaxation (−dP/dt) were measured.

### 2.4. Measurement of Blood Electrolytes

Whole blood levels of sodium, potassium, and calcium ion in rats 6 hours after LPS infusion were measured by an arterial blood gas analyzer (AVL OPTI Critical Care Analyzer; AVL Scientific Corp., Roswell, USA).

### 2.5. MPO Activity Test

MPO activity has been demonstrated to correlate with the number of neutrophils [20] and was used as an index of neutrophil accumulation in the heart. It was determined using an MPO assay kit (CytoStore, Calgary, Canada) by measuring the H_2_O_2_-dependent oxidation of O-dianisidine, according to the manufacturer's instructions. MPO activity is expressed as unit per mg protein (U/mg protein).

### 2.6. Western Blot Analysis

The left ventricular myocardium was isolated 6 hours after LPS administration, which was immediately frozen in liquid nitrogen, and stored at −80°C until processed. Detection of phospho-p65 and MIF by Western blotting was performed as described previously [15]. The primary antibodies in this experiment were mouse monoclonal anti-phospho-p65 (Epitomics, USA; 1 : 1000), and rabbit polyclonal anti-MIF (BioVision, USA; 1 : 1000).

### 2.7. Cardiomyocyte Isolation and Measurement of the Intracellular Calcium

Six hours after LPS administration, the heart was isolated. The methodology of tissue preparations and cardiomycytes isolation were followed and modified from previous studies [21, 22]. Intracellular calcium ([Ca^2+^]_i_) was recorded by an indo-1 fluorometric ratio technique. The fluorescent indicator indo-1 was loaded by incubating the myocytes of ventricle in sham, LPS, and LPS + HS groups at room temperature (25°C) for 20 to 30 minutes with 25 *μ*M of indo-1/AM (Sigma Chemical, St. Louis, MO). The Ca^2+^ transient was measured during a 2 Hz field-stimulation with 10-ms twice-threshold strength square-wave pulses. The fluorescence ratio data were processed and stored in a computer using software (OSP-SFCA; Olympus). Sarcoplasmic reticulum (SR) Ca^2+^ content was estimated by adding 20 mM caffeine after electric stimulation at 2 Hz for at least 30 s. The total SR Ca^2+^ content was measured from the amplitude of caffeine-induced Ca^2+^ transients.

### 2.8. Statistical Analysis

The data are expressed as means ± SEM. Statistical evaluation was performed with one-factor analysis of variance followed by the Newman-Keuls post hoc comparison test. A *P* value of less than 0.05 was deemed significant.

## 3. Results

### 3.1. Effects of HS on Hemodynamic Variables

**Figure 1:**
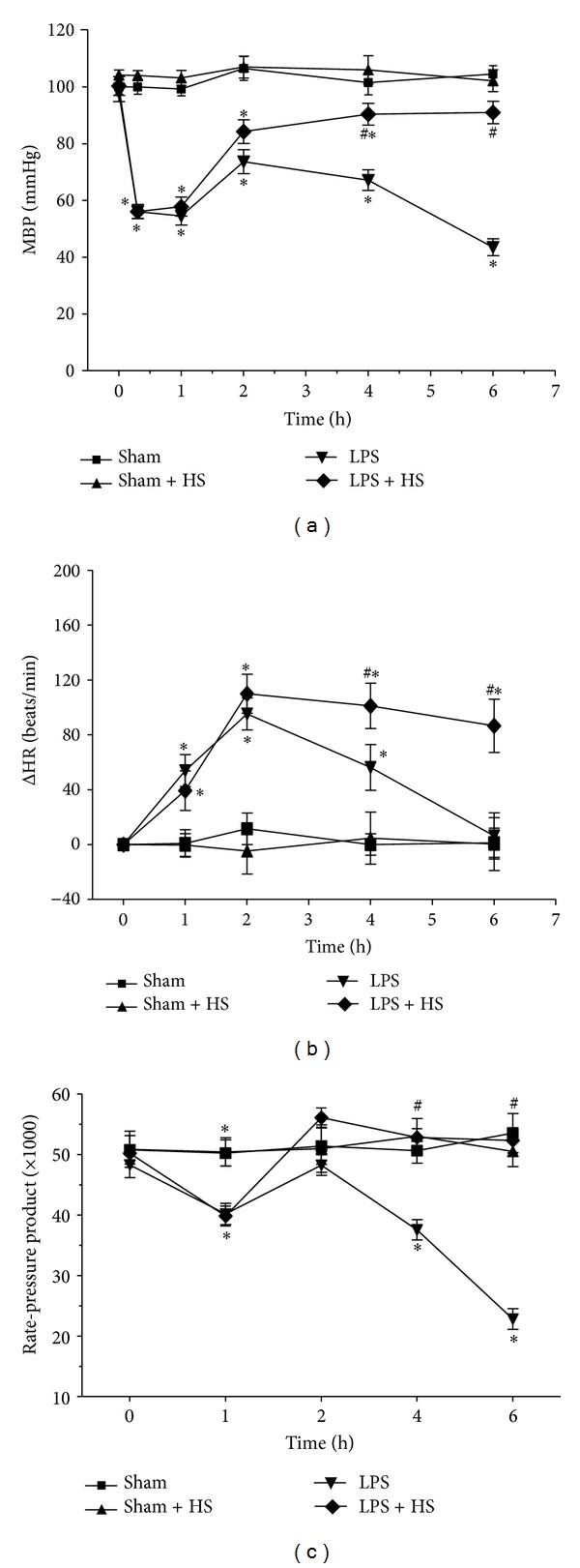
Effects of hypertonic saline (HS) on mean arterial blood pressure (a), changes in heart rate (b), and rate-pressure product (c) in conscious rats with sepsis-induced by LPS injection. *n* = 6–10. Values are expressed as mean ± SEM. *P < 0.05 versus the sham group; ^#^ *P* < 0.05 versus the LPS group.

The mean arterial blood pressure (MBP), heart rate, and rate-pressure product are shown in Figure 1. Rate-pressure product is provided by calculation using systolic blood pressure and heart rate and can reflect the cardiac work *in vivo* [23]. The basal MBP, heart rate, and rate-pressure product did not show significant differences. In sham and sham + HS groups, there were no significant changes in these variables throughout the experiment. In LPS group, MBP decreased gradually after LPS administration, which lasted until 1 h, and then progressively increased between 1 and 2 h, followed by a continued decrease between 2 and 6 h (Figure 1(a)). The MBP in LPS + HS group also initially decreased after LPS administration and recovered between 4 and 6 h, which is significantly higher than LPS group. After LPS administration, heart rate significantly elevated and peaked at 2 h, and then gradually decreased to basal level at 6 h. In LPS + HS group, the tachycardia caused by LPS lasted to 6 h, which was significantly higher than LPS group (Figure 1(b)). Furthermore, LPS challenge caused a marked reduction in rate-pressure product during 4–6 h. Posttreatment of HS markedly improved the reduced rate-pressure product caused by LPS (Figure 1(c)).

### 3.2. Effects of HS on Cardiac Contractile Dysfunction Caused by LPS

**Figure 2:**
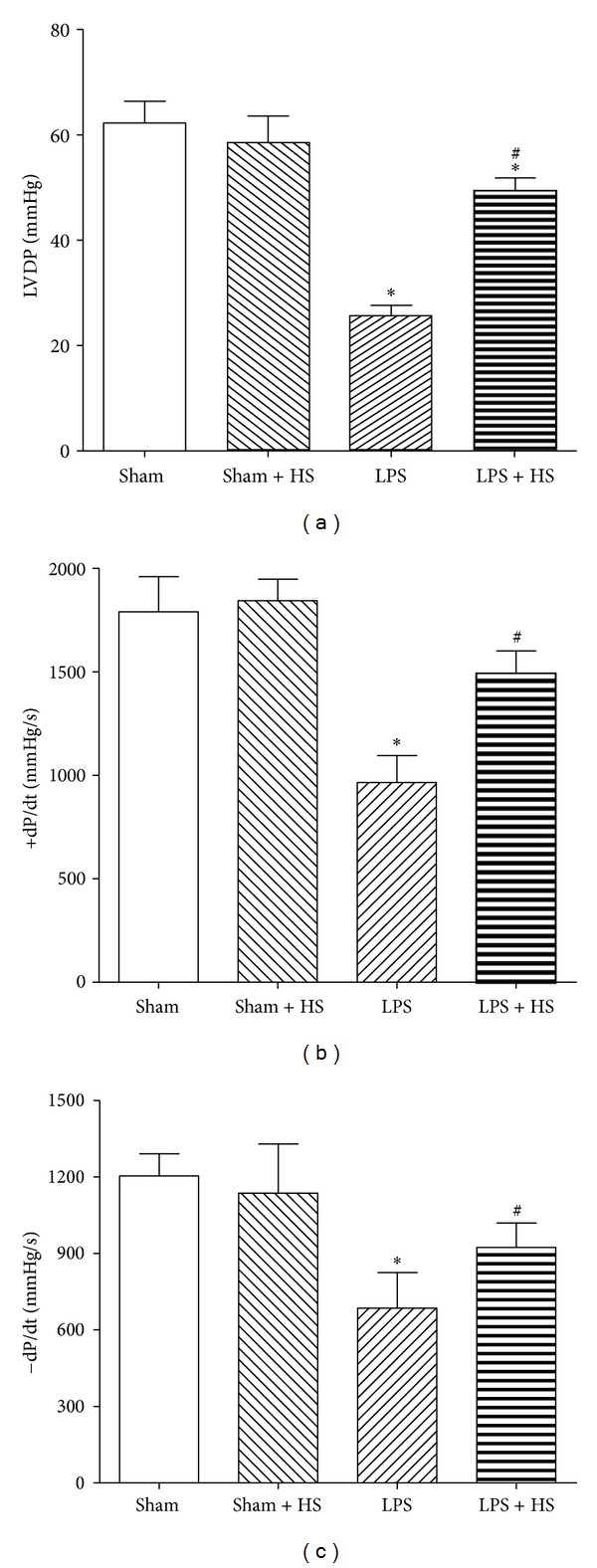
Effects of hypertonic saline (HS) on cardiac contractile dysfunction caused by LPS. (a) Left ventricular developed pressure (LVDP); (b) and (c) +dP/dt and −dP/dt in hearts 6 h after being subjected to LPS administration, *n* = 6–9. Data are given as mean ± SEM. *P < 0.05 versus the sham group, ^#^ *P* < 0.05 versus the LPS group.

The LVDP (Figure 2(a)) and average ±dP/dt (Figures 2(b) and 2(c)) were measured at 6 h after LPS administration, which was significantly reduced in LPS group compared with sham group (*P* < 0.05). After HS administration, LVDP and ±dP/dt significantly improved when compared with LPS group (*P* < 0.05). HS alone (sham + HS group) did not affect the cardiac contractile function compared with sham group.

### 3.3. Effects of HS on Liver and Renal Dysfunction and Cell Toxicity Caused by LPS

**Figure 3:**
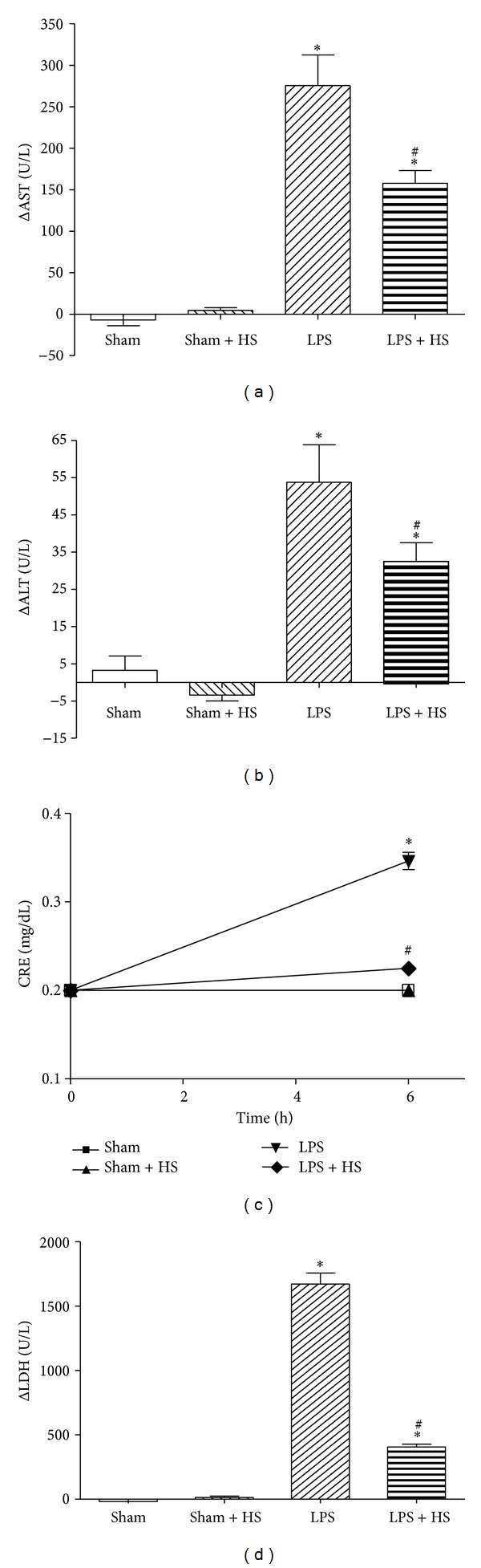
Effects of hypertonic saline (HS) on plasma levels of (a) alanine aminotransferase (ALT), (b) aspartate aminotransferase (AST), (c) creatinine (CRE), and (d) lactate dehydrogenase (LDH) in rats with sepsis-induced by LPS injection, *n* = 6–10. Values are expressed as mean ± SEM. *P < 0.05 versus the sham group; ^#^ *P* < 0.05 versus the LPS group.

The basal levels of AST, ALT, CRE, and LDH were not significantly different. LPS administration induced elevation of plasma levels of AST, ALT, CRE, and LDH at 6 h. The differences between 6 h levels and basal levels of AST, ALT, and LDH in the LPS group were significantly higher than sham group (Figures 3(a), 3(b), and 3(d)). HS treatment significantly decreased the elevation of AST, ALT, and LDH. The elevated CRE level caused by LPS also significantly attenuated after HS treatment (Figure 3(c)).

### 3.4. Effects of HS on MPO Activity in Ventricle after LPS Treatment

**Figure 4:**
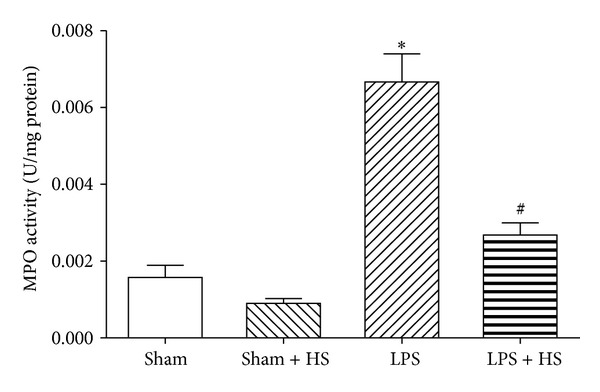
Effects of hypertonic saline (HS) on MPO activity in left ventricular myocardium of rats 6 h after being subjected to LPS administration, *n* = 6–8. Data are given as mean ± SEM. *P < 0.05 versus the sham group, ^#^ *P* < 0.05 versus the LPS group.

Treating sham rats with HS revealed a slight reduction in cardiac MPO activity (Figure 4). Six hours after LPS challenge, MPO activity increased by 4 folds compared with sham group (*P* < 0.05). HS treatment significantly suppressed MPO activity of LPS-challenged rats to the level similar to sham group (*P* > 0.05).

### 3.5. Effects of HS on Protein Expression in Rat Heart after LPS Treatment

**Figure 5:**
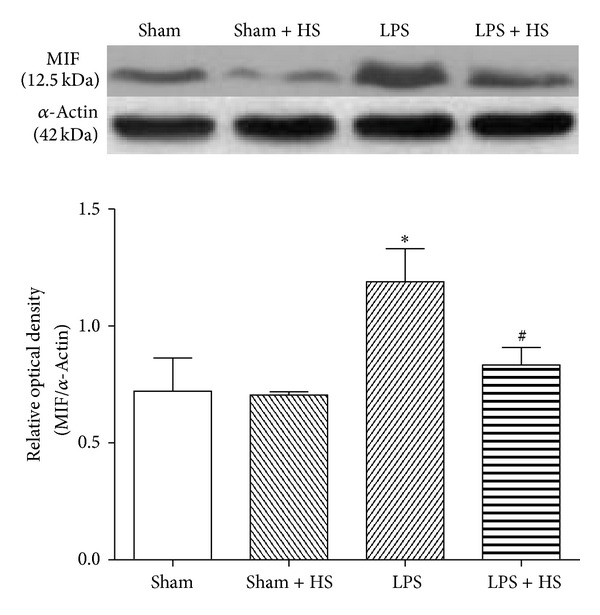
Effects of hypertonic saline (HS) on MIF protein expression in left ventricular myocardium of rats 6 h after being subjected to LPS administration. *n* = 6–9. Data are given as mean ± SEM. *P < 0.05 versus the sham group, ^#^ *P* < 0.05 versus the LPS group.

**Figure 6:**
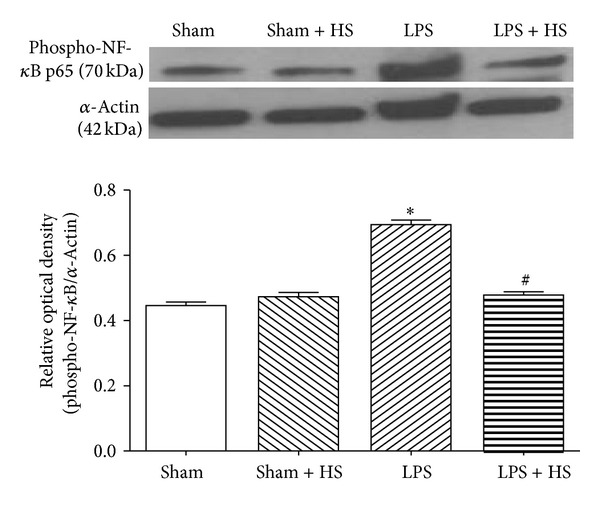
Effects of hypertonic saline (HS) on phospho-p65 protein expression in left ventricular myocardium of rats 6 h after being subjected to LPS administration, *n* = 6–10. Data are given as mean ± SEM. *P < 0.05 versus the sham group, ^#^ *P* < 0.05 versus the LPS group.

The protein expression of MIF (Figure 5) and phospho-p65 (Figure 6) was significantly elevated after 6 h LPS administration (*P* < 0.05). HS treatment significantly suppressed LPS-induced increases in MIF and phospho-p65 protein expression (*P* < 0.05).

### 3.6. Effects of HS on Ion Concentrations in Blood after LPS Treatment

**Figure 7:**
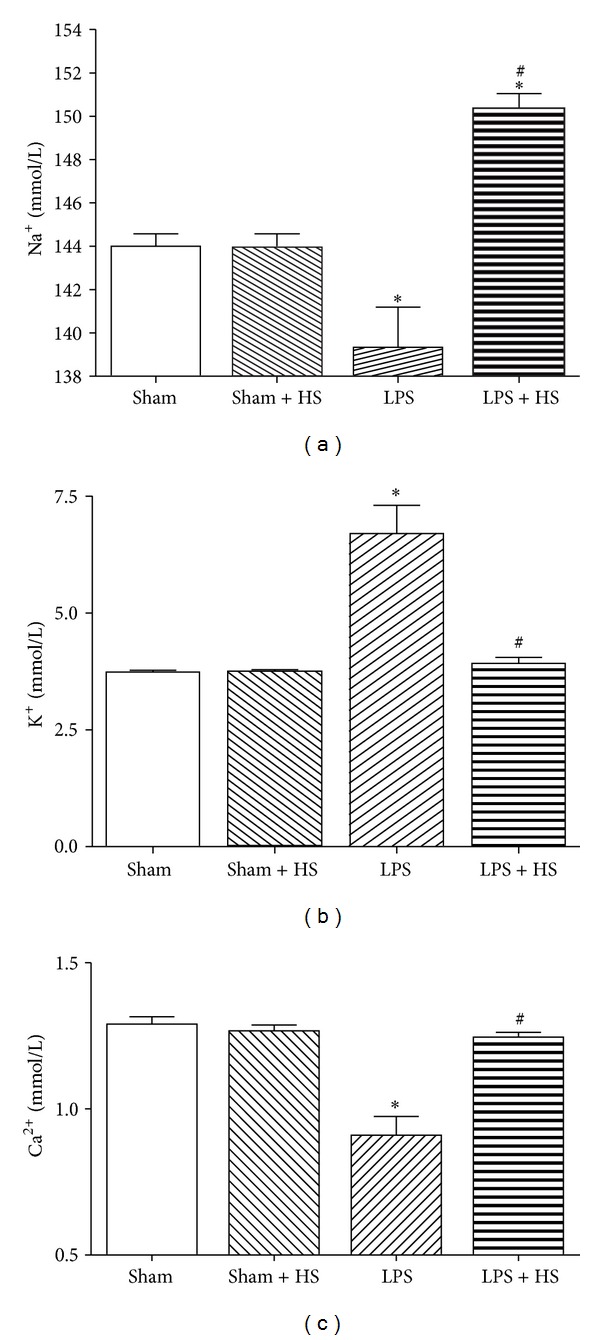
Effects of hypertonic saline (HS) on whole blood levels of (a) sodium ion, (b) potassium ion, and (c) calcium ion in rats with sepsis, *n* = 3–5. Values are expressed as mean ± SEM. *P < 0.05 versus the sham group; ^#^ *P* < 0.05 versus the LPS group.

Treating HS with sham-operated rats did not show elevation of Na^+^, K^+^, and Ca^2+^ concentrations in blood. LPS administration can cause a significant reduction in Na^+^ concentration at 6 h compared with sham group. A dramatic elevation of Na^+^ concentration was found in LPS + HS group compared with sham and LPS groups (Figure 7(a)). By contrast, LPS resulted in marked increase of K^+^ concentration when compared with sham group. Receiving HS treatment, LPS-treated rats showed marked reduction in K^+^ concentration to levels similar to those of sham group (Figure 7(b)). Moreover, LPS resulted in a significant reduction in Ca^2+^ concentration in blood at 6 h compared with sham group, which was reversed by HS treatment (Figure 7(c)).

### 3.7. Effects of HS on Intracellular Ca^2+^ Concentration in Rat Heart after LPS Treatment

**Figure 8:**
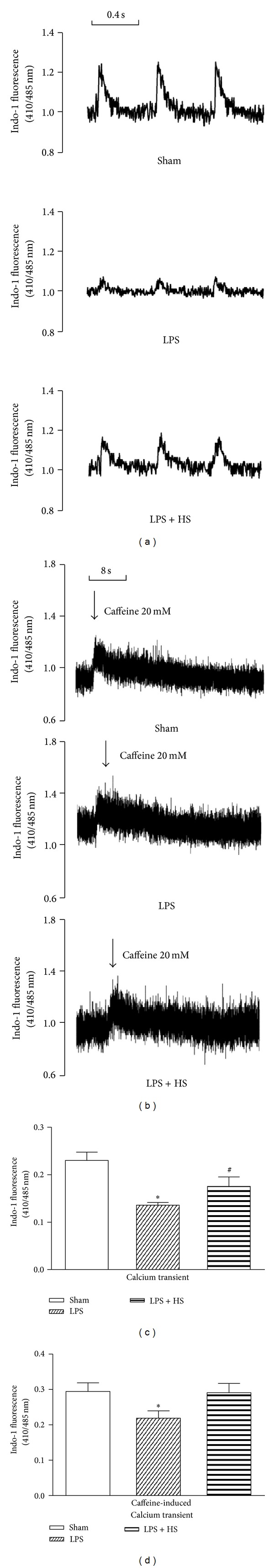
Effects of hypertonic saline (HS) on Ca^2+^ transient of cardiomyocytes in rats with sepsis. Panels (a) and (c) show the tracings and the average of electrical-stimulation Ca^2+^ transient. Panels (b) and (d) show the tracings and the average of caffeine-induced Ca^2+^ transient; *n* = 9 in each group. Values are expressed as mean ± SEM. *P < 0.05 versus the sham group; ^#^ *P* < 0.05 versus the LPS group.

As shown in Figure 8, the electrical-stimulation Ca^2+^ transient of ventricular cardiomyocytes significantly reduced 6 h after LPS challenge, which was significantly recovered by HS treatment. Similarly, the caffeine-induced Ca^2+^ transient was significantly reduced 6 h after LPS challenge. However, HS did not significantly affect this change.

## 4. Discussion

The present study demonstrated that posttreatment with HS can ameliorate circulatory failure including hypotension and cardiac dysfunction caused by LPS-induced sepsis in a conscious rat model. The cardioprotective effect of HS is associated with improvement of [Ca^2+^]_i_ handling process, attenuation of neutrophil infiltration, MIF protein expression, and transcription factor NF-*κ*B activation in myocardium.

Shih et al. [6] demonstrated similar results in peritonitis-induced septic shock, which are related to the anti-inflammatory and antioxidant effect of HS. We further demonstrated that posttreatment with HS significantly showed cardioprotective effects, which were evidenced by increased contractile function and maintenance of compensatory tachycardia 6 h after LPS challenge. HS provides an intravascular hypertonic environment, leading to increase of the plasma volume, which may contribute to improve the cardiac output, blood flow, and multiple organ function.

It has been shown that MPO in myocardial tissue significantly increased in LPS-induced sepsis [24]. MPO will be released when neutrophils infiltrate into the organs. Ninety percent of MPO released from neutrophils. Measuring the content of MPO can speculate neutrophil infiltration in organs and tissues [25]. MPO expression in left ventricular myocytes was significantly higher in failed hearts, suggesting that overexpression of MPO caused damages to the cardiac function [26]. HS administration significantly reduced MPO accumulation in the myocardial tissue, indicating that neutrophil infiltration was reduced by HS. This anti-inflammatory effect is likely to contribute to the cardioprotection of HS.

Plasma MIF content peaks in early sepsis [27]. Overexpression of MIF protein in sepsis causes cardiac dysfunction [11]. MIF antibody treatment can preserve the cardiac function of mice in sepsis [13]. In this study, HS significantly reduced MIF protein expression in myocardium and maintained cardiac function, suggesting that the inhibitory effect on MIF production contributes to the cardioprotection of HS in sepsis. Inhibition of MIF can suppress NF-*κ*B activation, whereas inhibition of NF-*κ*B activity significantly attenuates MIF performance [28]. In this study, HS can reduce NF-*κ*B activation in cardiac tissue (Figure 6). Therefore, we suggest that, via suppression of neutrophil infiltration into, myocardium, HS attenuates inflammation-related responses, for example, MIF release by immune cells and NF-*κ*B activation in cardiomyocytes during sepsis. The inhibitory effect on NF-*κ*B activation contributes to decrease in MIF production, by which cardiac contractile function was protected.

Clinical sepsis patients often have low blood sodium phenomena coincide [29]. In the present study, we also found that sodium ion concentration in blood of LPS group is significantly lower than sham group. The underlying mechanism is still uncertain. Hyponatremia may be due to cytokines-induced downregulation of angiotensin II type 1 receptors, resulting in impaired regulation of sodium and water balance by aldosterone and leading to sodium and water loss [30]. After five hours of HS post-treatment, the sodium ion concentration about 153 mmol/L in LPS + HS group, which was significantly higher than another groups. In a previous study, hypernatremic phenomenon has been shown to suppress human phagocytic activity and superoxide anion production [31]. HS supplement can increase the sodium concentration and may reduce neutrophil activation in LPS-induced sepsis.

Furthermore, HS reverses hypocalcemia induced by LPS (Figure 7(c)). A similar result has been demonstrated in a cecal ligation and puncture-induced peritonitis of rats [32]. Sepsis can induce hypocalcemia, which is associated with intracellular Ca^2+^ accumulation [33]. Elevated intracellular Ca^2+^ levels have been reported to activate Ca^2+^-dependent proteolytic enzymes, leading to tissue damage in sepsis [34]. In a previous study, HS dextran demonstrated to attenuate diastolic levels of [Ca^2+^]_i_ in cardiomyocyte after burn complicated with sepsis in late stage [35]. In this study, the electrically-induced [Ca^2+^]_i_ transient was measured to observe the influx of Ca^2+^ via the L-type Ca^2+^ channel upon electrical stimulation, which then triggers release of Ca^2+^ from the sarcoplasmic reticulum, leading to muscle contraction. The electrically induced [Ca^2+^]_i_ transient is directly related to contractility [36]. HS supplement in early sepsis can improve the amplitude of intracellular Ca^2+^ transient, which was significantly reduced in our acute sepsis model, indicating the intracellular Ca^2+^ handling process was recovered (Figure 8). We also found the caffeine-induced Ca^2+^ transient significantly reduced during sepsis, indicating the Ca^2+^ content of SR decreased, leading to a reduction in Ca^2+^ release from SR and the decrease in contractility. The Ca^2+^ content of SR was elevated after HS treatment. Therefore, HS may improve cardiac contractile function via maintenance of intracellular Ca^2+^ homeostasis. On the other hand, proinflammatory cytokines, such as TNF-*α* and IL-1*β*, have been implicated in ventricular dysfunction associated with sepsis [37, 38]. TNF-*α* and IL-1*β* increase the SR Ca^2+^ leak from the SR, which contributes to the depressed Ca^2+^ transient and contractility [39]. Therefore, the anti-inflammatory effect of HS can contribute to maintain [Ca^2+^]_i_ handling capacity to improve the cardiac contractile function.

## 5. Conclusion

HS improved cardiac contractile function and Ca^2+^ homeostasis in sepsis, which contribute to ameliorate circulatory failure and to maintain multiple organ function. Attenuation of neutrophil infiltration, suppression of NF-*κ*B activation, and reduced MIF production in myocardium are associated with the cardioprotective effect of HS in sepsis.
